# Use of Herbal Medicine by Pregnant Women: What Physicians Need to Know

**DOI:** 10.3389/fphar.2019.01483

**Published:** 2020-01-09

**Authors:** Sílvia M. Illamola, Ogochukwu U. Amaeze, Lubov V. Krepkova, Angela K. Birnbaum, Ashwin Karanam, Kathleen M. Job, Valentina V. Bortnikova, Catherine M.T. Sherwin, Elena Y. Enioutina

**Affiliations:** ^1^Division of Clinical Pharmacology, Department of Pediatrics, University of Utah School of Medicine, Salt Lake City, UT, United States; ^2^Department of Experimental and Clinical Pharmacology, College of Pharmacy, University of Minnesota, Minneapolis, MN, United States; ^3^Department of Clinical Pharmacy and Biopharmacy, Faculty of Pharmacy, University of Lagos, Lagos, Nigeria; ^4^Center of Medicine, All-Russian Research Institute of Medicinal and Aromatic Plants (VILAR), Moscow, Russia; ^5^Department of Pharmacotherapy, College of Pharmacy, University of Utah, Salt Lake City, UT, United States; ^6^Department of Pathology, School of Medicine, University of Utah, Salt Lake City, UT, United States; ^7^Pharmaceutics & Pharmaceutical Chemistry, College of Pharmacy, University of Utah, Salt Lake City, UT, United States

**Keywords:** pregnancy, herbal medicine, herbal-drug interaction, efficacy, safety

## Abstract

About 80% of the consumers worldwide use herbal medicine (HMs) or other natural products. The percentage may vary significantly (7%–55%) among pregnant women, depending upon social status, ethnicity, and cultural traditions. This manuscript discusses the most common HMs used by pregnant women, and the potential interactions of HMs with conventional drugs in some medical conditions that occur during pregnancy (e.g., hypertension, asthma, epilepsy). It also includes an examination of the characteristics of pregnant HM consumers, the primary conditions for which HMs are taken, and a discussion related to the potential toxicity of HMs taken during pregnancy. Many cultures have used HMs in pregnancy to improve wellbeing of the mother and/or baby, or to help decrease nausea and vomiting, treat infection, ease gastrointestinal problems, prepare for labor, induce labor, or ease labor pains. One of the reasons why pregnant women use HMs is an assumption that HMs are safer than conventional medicine. However, for pregnant women with pre-existing conditions like epilepsy and asthma, supplementation of conventional treatment with HMs may further complicate their care. The use of HMs is frequently not reported to healthcare professionals. Providers are often not questioning HM use, despite little being known about the HM safety and HM-drug interactions during pregnancy. This lack of knowledge on potential toxicity and the ability to interact with conventional treatments may impact both mother and fetus. There is a need for education of women and their healthcare professionals to move away from the idea of HMs not being harmful. Healthcare professionals need to question women on whether they use any HMs or natural products during pregnancy, especially when conventional treatment is less efficient and/or adverse events have occurred as herbal-drug interactions could be the reason for these observations. Additionally, more preclinical and clinical studies are needed to evaluate HM efficacy and toxicity.

## Introduction

The use of herbal medicines (HMs) (World Health Organization, Traditional, complementary and integrative medicine. Definitions, 2013), including medicinal herbs as botanical drugs, teas, dietary supplements (United States Congress), or indigenous formulation containing herbs, has increased significantly in the last two decades (Barnes, 2016; Sammons et al., 2016; Teng et al., 2016; Enioutina et al., 2017). Women are the primary consumers of HMs, and usually continue use during pregnancy (Dugoua, 2010). The prevalence of HM use during pregnancy ranges from 7% to 55% (Tiran, 2003), depending on consumers geographic location, ethnicity, cultural traditions, and social status (Dugoua, 2010). For example, 34% of Australian (Frawley et al., 2013), ~50% of European Union (Lapi et al., 2010; Holst et al., 2011), and 6%–9% of the USA and Canadian (Moussally et al., 2009; Louik et al., 2010) pregnant women are using HMs. Pregnant women usually perceive herbal products as a safe, natural alternative to conventional drugs (Frawley et al., 2015) and often use them to improve their wellbeing or for the treatment of non-life threating conditions (e.g., nausea, constipation).

A number of HMs or dietary supplements, such as uterine stimulants and labor inducers {e.g., Black cohosh (*Actaea racemosa* L.), and Blue cohosh [*Caulophyllum thalictroides* (L.) Michx.]} (Dugoua et al., 2006; Dugoua et al., 2008; Frawley et al., 2015), have a long, documented history of use. However, while the adverse effects of these herbal treatments are documented (Smeriglio et al., 2014), data on their safety during pregnancy is generally insufficient. In most countries, HMs are available over the counter, making them very accessible. HMs can represent a problem when self-prescribed by pregnant women along clinician-prescribed conventional drugs (Low Dog, 2009). As patients do not recognize them as potentially harmful compounds, HM use is not always reported to healthcare professionals (Adams, 2011).

The main purpose of this scoping review is to inform healthcare professionals regarding HMs commonly used by pregnant women and potential interactions of these HMs with conventional drugs used for the treatment of some preexisting medical conditions or those that occur during pregnancy (e.g., hypertension, asthma, epilepsy). Hypertensive disorders during pregnancy, including chronic hypertension, gestational hypertension, preeclampsia, and chronic hypertension with superimposed preeclampsia, represent about 10% of pregnancy complications in the USA (Lai et al., 2017). Asthma is a common preexisting condition affecting about 9% of pregnant women (Kwon et al., 2006). Epilepsy is another common preexisting disease in pregnant women. It has been reported that use of antiepileptic drugs (AEDs) by pregnant women may be associated with an increase in adverse outcomes such as miscarriage, antepartum, and post-partum hemorrhages (Viale et al., 2015). This manuscript also includes an examination of the characteristics of pregnant HM consumers, the primary conditions for which HMs are taken, and a discussion related to the potential toxicity of HMs taken during pregnancy.

## Methods

The study undertaken by our group was a scoping review of the literature aimed at identifying literature describing 1) medicinal herb and traditional medicine use by women during pregnancy and 2) gaps in the pre-clinical and clinical research associated with the use of medicinal herbs and traditional remedies by pregnant women. A comprehensive search was conducted by six reviewers in two electronic databases (PubMed and Embase) and relevant medicinal herbs websites for articles published between January 1983 and December 2018. Searches were conducted using a base of keywords: medicinal herbs, phytomedicine, traditional medicine, conventional drugs, herb-drug interaction, adverse drug (herb) reactions, hypertension treatment, asthma treatment, epilepsy treatment, pregnancy, and pregnant women. Combinations and variations of keywords were also used. Bibliographies of all relevant eligible articles were reviewed for further potential references. Pre-clinical animal, *in vitro* study, and clinical trial data reported in the selected articles were categorized into thematic groups related to the study objectives.

## Consumption of Hms by Pregnant Women

Multiple studies over the last decade reveal that pregnant women may take a variety of HMs as crude herbal preparations, herbal extracts, finished and labeled medicinal products of herbal origin as well as dietary supplements consisting of proprietary blends of HMs, vitamins, and minerals. The most commonly used HMs were ginger (*Zingiber officinale* Roscoe*)*, chamomile (*Matricaria chamomilla* L.), peppermint (*Mentha* piperita L.), Echinacea [*Echinacea purpurea* (L.) Moench], cranberry (*Vaccinium oxycoccus* L. and *Vaccinium macrocarpum* L.), garlic (*Allium sativum* L.), raspberry (*Rubus idaeus* L.), valerian (*Valeriana officinalis* L.), fenugreek (*Trigonella* foenum*-*graecum L.), fennel (*Foeniculum vulgare* Mill.), herbal blends, and teas including green and black teas [*Camellia sinensis* (L.) Kuntze] (Adams et al., 2009; Hall et al., 2011; Kennedy et al., 2013; John and Shantakumari, 2015; Teni et al., 2017). Herbal products (e.g., ginger, garlic, and various herbal teas) were frequently used by pregnant women worldwide, while others were region-specific. For example, motherwort (*Leonurus cardiaca* L.), centaury (*Centaurium erythraea* Rafn.), and lovage (*Levisticum officinale* W.D.J.Koch) were mostly used by women from Eastern European countries while Uva ursi [*Arctostaphylos uva-ursi* (L.) Spreng.] was used by women living in Western and Eastern European countries (Kennedy et al., 2013). Floradix^®^ IRON + HERBS, ginseng, and valerian were often used by Swedish women during pregnancy (Holst et al., 2008).

The indications for the use of HMs during pregnancy may vary and can be either mother- or child-related. HMs may be used sometimes as a part of maternal care to treat pregnancy-related problems, and often to improve the well-being of the mother or unborn child. The most commonly reported indications were nausea and vomiting, urinary tract infections (UTIs), preparation for and/or facilitation of labor, cold or flu, gastrointestinal issues, pain conditions, improvement of fetal outcomes, prevention of miscarriage, anxiety, health maintenance, and edema (Hall et al., 2011; Kennedy et al., 2013; John and Shantakumari, 2015; Teni et al., 2017). The most common herbal products consumed during pregnancy and their indications are described in **Table 1**.

**Table 1:** Commonly used herbal medicines (HMs) by pregnant women worldwide.

Indications	Medicinal herbs
Morning sickness, nausea, vomiting	Ginger (*Zingiber officinale* Roscoe), Peppermint (*Mentha piperita* L.), Herbal teas, Raspberry (*Rubus idaeus* L.), Bishop’s weed (*Aegopodium podagraria* L.), Garlic (*Allium sativum* L.), Anise (*Pimpinella anisum* L.)
Cold and flu	Madder (*Rubia tinctorum* L.), Anise (*Pimpinella anisum* L.), Golden buttons (*Matricaria aurea* (Loefl.) Sch. Bip.), Wild thyme (*Origanum syriacum* L.), Liquorice (*Glycyrrhiza glabra* L.), Borage (*Borago officinalis* L.), Chamomille (*Matricaria chamomilla* L.), Ginger (*Zingiber officinale* Roscoe), Echinacea (*Echinacea purpurea* L.), Eucalyptus (*Eucalyptus* spp.), Rosehip (*Rosa* spp.)
Pain (gastralgia and other types of pain)	Verbena triphylla (*Aloysia citriodora* Palau), Anise (*Pimpinella anisum* L.), Cumin (*Cuminum cyminum* L.), Fennel (*Foeniculum vulgare Mill*.), Golden buttons [*Matricaria aurea* (Loefl.) Sch. Bip.], Peppermint (*Mentha piperita* L.), Wild thyme (*Origanum syriacum* L.), Sage (*Salvia officinalis* L.), Cinnamon (*Cinnamomum verum* J. Presl), Black cumin (*Nigella ciliaris* DC.), Chamomille (*Matricaria chamomilla* L.)
Anxiety, stress	Anise (*Pimpinella anisum* L.), Peppermint (*Mentha piperita* L.), Chamomille (*Matricaria chamomilla* L.), Thyme (*Thymus vulgaris* L.), Rosemary (*Rosmarinus officinalis* L.), Valerian (*Valeriana officinalis* L.)
Gastrointestinal disorders, constipation, flatulence	Fennel (*Foeniculum vulgare* Mill.), Anise (*Pimpinella anisum* L.), Borage (*Borago officinalis* L.), Chamomille (*Matricaria chamomilla* L.), Ginger (*Zingiber officinale* Roscoe), Peppermint (*Mentha piperita* L.), Senna (*Cassia senna*), Green tea (*Camellia sinensis*), Cinnamon (*Cinnamomum verum J. Presl*)
Edema	Turmeric (*Curcuma longa L*.), Fennel (*Foeniculum vulgare Mill*.)
Urinary tract infection	Cranberry (*Vaccinium macrocarpon* L.), Bearberry [*Arctostaphylos uva-ursi*) (L.) Spreng.], Parsley [*Petroselinum crispum* (Mill.) Fuss], Fenugreek (*Trigonella foenum-graecum L*.), Rosemary (*Rosmarinus officinalis L*.), Peppermint (*Mentha piperita L*.), Sage (*Salvia officinalis L*.)
Labor preparation, facilitation and induction	Rooibos [*Aspalathus linearis* (Burm.f.) R. Dahlgren] tea, coconut (*Coco nucifera* L.) oil, Date palm (*Phoenix dactylifera* L.), Golden buttons [*Matricaria aurea (Loefl.) Sch. Bip*.], Watercress (Nasturtium officinale W.T. Aiton), Cinnamon (*Cinnamomum verum J. Presl*), Fenugreek (*Trigonella foenum-graecum*), Rosemary (*Rosmarinus officinalis L*.), Raspberry (*Rubus idaeus L*.), *Cannabis sativa* L., Evening primrose (*Oenothera biennis L.)*
Milk production and secretion	Madder (*Rubia tinctorum* L.), Caraway (*Carum carvi* L.), Fenugreek (*Trigonella foenum-graecum L*.), Cinnamon (*Cinnamomum verum J. Presl*), Cumin (*Cuminum cyminum L*.), Fennel (*Foeniculum vulgare* Mill.)
Fetal health promotion	Gingko (*Gingko biloba*), Ganoderma (*Ganoderma lucidum*), Sage (*Salvia fruticosa*), Olibanum (*Boswellia serrata* Triana & Planch.)
Anemia	Spinach (*Spinacia oleracea* L.), Fenugreek (*Foeniculum vulgare Mill*.), Cinnamon (*Cinnamomum verum* J. Presl), Rosehip (*Rosa* spp.)

While the use of HMs for the above described indications is not country specific, some indications for HM use were more prominent within particular regions. For example, pregnant women in India and Ghana were more likely to use HMs to prevent miscarriage and improve the health status of the unborn baby than their counterparts in the developed nations (Adusi-Poku et al., 2015; Bhatt, 2016). Common fetal-related indications were an improvement of fetal physical and mental growth (Rahman et al., 2008; Adusi-Poku et al., 2015; Mugomeri et al., 2015; Bhatt et al., 2016; Dika et al., 2017), prevention of neonatal hyperbilirubinemia (Tabatabaee, 2011), or even protecting the unborn baby from evil (Maputle et al., 2015). Remarkably, some indications were firmly ingrained in the cultural beliefs of women. For instance, most women in Lesotho used HMs during pregnancy simply because it was a tradition (Mugomeri et al., 2015). Similarly, in Zimbabwe, the use of antenatal HMs was carried over from previous generations. Pregnant women were culturally obliged to use traditional treatments, sometimes against their personal preference (Panganai and Shumba, 2016).

The use of indigenous medicinal plants as a part of antenatal care was common in developing nations, such as in Africa and Asia (Mureyi et al., 2012; Al-Ramahi et al., 2013; Elkhoudri et al., 2016; Maputle et al., 2015; Tang et al., 2016; Onyiapat et al., 2017). Such medicinal plants were typically consumed in a crude form, or as infusions, decoctions, and tinctures. The “*Isihlambezo*” decoction for instance, was used as a tonic by pregnant Zulu women in South Africa during the last trimester of pregnancy (Varga and Veale, 1997; Brookes, 2018). “*Isihlambezo*,” comprised of several medicinal plants, was strongly believed to “promote a favorable course of pregnancy and facilitate quick and uncomplicated labor” (Veale et al., 1992; Varga and Veale, 1997; Naidu, 2014; Brookes, 2018). The four most frequently cited components of this decoction are *Clivia miniata* (Lindl.) Verschaff., *Agapanthus africanus* (L.) Hoffmanns, *Pentanisia prunelloides* (Klotzsch ex Eckl. & Zeyh.) Walp., and *Gunnera perpensa* L. (Brookes, 2018). The recipes depend on a number of factors including the traditional healer preferences, the woman’s health status, and many other factors (Veale et al., 1992). This decoction possesses direct uterotonic activity. When used at a high dose, it may cause excessive uterine stimulation leading to adverse reactions. The adverse effects linked to “*Isihlambezo*” formulation include low neonatal birth-weights, fetal meconium staining, which was a sign of possible fetal distress and hypoxia, and fatal uterine rupture (Veale et al., 1998). The “*Mwanamphepo*” preparation, which contains several plants [e.g., *Ampelocissus obtusata* (Welw. ex Baker) Planch., *Cyphostemma hildebrandtii* (Gilg) Desc. ex Wild & R.B.Drumm, and *Cissus verticillata* (L.) Nicolson & C.E.Jarvis], was commonly used in Malawi to induce labor (Maliwichi-Nyirenda and Maliwichi, 2010). It was allegedly mixed with porridge and consumed by pregnant women before labor (Maliwichi-Nyirenda and Maliwichi, 2010). The self-reported use of ‘*Mwanamphepo*” preparation during pregnancy in rural Malawi was associated with increased morbidity of mothers and increased mortality and morbidity of neonates (Zamawe et al., 2018). In Korea, the herbal formulation “*Anjeonicheon-tang*” containing *Atractylodes macrocephala* Koidzumi white rhizoma, *Glycyrrhiza glabra* L. rhizoma, *Panax ginseng* C.A.Mey. rhizoma, and other medicinal plants was frequently used during pregnancy for pregnancy-related symptoms, especially hyperemesis gravidarum (Jo et al., 2016). “*An-Tai-Yin*” containing numerous medicinal herbs in formulation is consumed by pregnant women in Taiwan (Chuang et al., 2009). *An-Tai-Yin* use was associated with an increased risk of congenital malformations of the fetal eye, musculoskeletal, and connective tissues (Chuang et al., 2006).

It appears that HM use was prevalent in the first and third trimesters of pregnancy (John and Shantakumari, 2015; Pallivalapila et al., 2015). HM was usually used in the first trimester for prevention and/or treatment of early pregnancy-related issues such as nausea, vomiting, and gastrointestinal disorders. During the third trimester, they were commonly used to prepare the uterus for labor and ease child delivery (Mureyi et al., 2012; Nyeko et al., 2016; Onyiapat et al., 2017). Nonetheless, it was not uncommon for women to use HMs throughout all trimesters of pregnancy, usually with the intent to improve the well-being of the mother and unborn child. No particular trends have been revealed by researchers (Nordeng and Havnen, 2004; Rahman et al., 2008; Tabatabaee, 2011; Tang et al., 2016). In some cases, the women simply continued using the herbal products until they “felt better” (Nergard et al., 2015).

The majority of pregnant women preferring complementary or alternative medicine containing HMs were married women with secondary schooling or university degrees (Zaffani et al., 2006; Adams et al., 2009; Kennedy et al., 2013; Kennedy et al., 2016). These HMs users were nonsmokers over 30 years old with higher incomes and a prior history of the HM use (Forster et al., 2006; Adams et al., 2009; Kennedy et al., 2013; Kennedy et al., 2016). In Eastern European countries, HM use was more prevalent among younger pregnant women (less than 20 years) (Kennedy et al., 2013). In African and Asian regions, women who use HMs during pregnancy were less than 30 years old, with little or no formal education, of low socioeconomic status, and living in rural areas far from public health facilities (John and Shantakumari, 2015; Teni et al., 2017).

The majority of pregnant women relied extensively on family members, relatives, and friends in their decisions to use HMs during pregnancy (Kennedy et al., 2013; John and Shantakumari, 2015). Other essential referral sources, particularly in Africa, were traditional birth attendants, local herb sellers, and herbalists (Teni et al., 2017). In developed nations, the media was one of the supporting sources in the decision regarding HM use during pregnancy (Hall et al., 2011). Pregnant women from the Eastern European countries frequently made their decisions based on advice of a treating physician (Kennedy et al., 2013). Concomitant consumption of HMs and conventional drugs by pregnant women is quite common worldwide (Holst et al., 2008; Tamuno et al., 2010; Holst et al., 2011; Tabatabaee, 2011; Nyeko et al., 2016). The simultaneous use of HMs and conventional drugs may result in a decrease of drug efficacy or toxicity resulting from herb-drug interactions. However, women often withhold this information on HM use from their healthcare professional because the use of HMs is not part of conventional treatment.

Our review of the literature has shown that while use of HMs by pregnant women may vary depending on region, cultural traditions, age, and education, the majority of pregnant women actively use various HMs and traditional remedies to prevent or treat conditions associated with pregnancy, to ease labor, and also to improve baby development after labor. This urgently necessitates the exploration of HMs and traditional herbal formulations for their safety and efficacy in pregnant animals or potentially women taking this HMs.

## Efficacy and Safety of HMs Used by Pregnant Women

The information on safety and efficacy of medicinal herbs generally comes from the experience of past generations. Preclinical and clinical studies completed in the last two to three decades have provided scientific evidence of the efficacy and safety of some medicinal herbs and herbal formulations.

Pre-clinical in vivo and in vitro studies describing potential embryotoxicity have been conducted for certain HMs. An extensive search conducted by Jurgens in 2003 revealed a few studies that scientifically evaluated safety of HMs, including blue cohosh [C. thalictroides (L.) Michx.], ginger (*Z. officinale* Roscoe), ginkgo (Ginkgo biloba L.), Siberian ginseng [*Eleutherococcus senticosus* (Rupr. & Maxim.) Maxim.], phytoestrogens, pyrrolizidine alkaloids, scullcap (*Scutellaria lateriflora* L.), St. John’s Wort (*Hypericum perforatum* L.), and valerian (*V. officinalis* L.) (Jurgens, 2003). Ginkgo was the only HM with no teratogenic or embryotoxic effects reported. The use of the other HMs was associated with embryotoxicity.

An *in vitro* study of the effects of ginsenosides, the bioactive component of ginseng, on the developing embryo during the critical period of organogenesis demonstrated that they exert direct teratogenic effects on rat embryos (Chan et al., 2003). Veale et al. (1998) studied the effects of several plant extracts commonly used as ethnic herbal oxytocics: *C. miniata* (Lindl.) Verschaff. (umayimi), *A. africanus* (L.) Hoffmanns (ubani), *P. prunelloides* (Klotzsch ex Eckl. & Zeyh) Walp (icishamlilo), *G. perpensa* L. (ugobo), *Rhoicissus tridentata* (L.f.) Wild & R.B.Drumm. (isinwazi), and *Combretum erythrophyllum* (Burch.) Sond. and *Combretum kraussii* Hochst. (umdubu) for their embryotoxicity. Based on results from an isolated rat uterus model, the authors concluded that the oxytocic activity of these plants can lead to fetal hypoxia and premature delivery as a result of uterine hyperstimulation. Mahmoudian et al. (Mahmoudian et al., 2012) investigated the effects of valerian (*V. officinalis* L.) extract on brain development and function in the brain tissues of murine fetuses. They found that the use of valerian during mid-gestation caused a significant decrease in zinc level in the fetal brain, an essential element affecting brain development. In our opinion, the use of valerian preparations should be limited during pregnancy. The treatment of pregnant rats with of thermopsis (*Thermopsis* lanceolata R.Br.), an HM with expectorant properties, resulted in a decrease of the body weight and craniocaudal measurements of 20-day old fetuses (Krepkova et al., 2016). Thermopsis did not affect the survival rate of pups in the postpartum period, but altered vestibular functions, motor coordination, and emotional-motor behaviors of pups born from mothers exposed to HM. Therefore, thermopsis and preparations containing thermopsis should not be recommend for the use during pregnancy.

The above reviewed studies have shown that HMs may possess embryotoxic, teratogenic effects, or may stimulate premature labor in preclinical studies. It is not known if these effects are also extant in humans. This explains why there are very few studies investigating efficacy and safety of HMs in pregnant women. Knowing that pre-clinical studies of HMs demonstrate embryotoxic effects and/or defects in offspring development, in our opinion, it would be unethical to do prospective studies; however, review of retrospective clinical studies would be helpful to identify clinically relevant herb-related birth defects.

Leung and colleagues performed a retrospective study comparing the differences in the neonatal development of babies born from pregnant women who took either HMs or pharmaceutical products for the treatment of flu and some chronic medical problems, as well as for weight reduction, menstrual problems, and infections (Leung et al., 2002). The prevalence of congenital fetal abnormalities in the group of women who took HMs was higher, but not statistically significant, than that in the group of women who took pharmaceutical products. There were neither differences in the proportion of silent miscarriages nor in the presence of abnormal karyotypes between the two groups. Although the authors did not find differences in the teratogenicity associated with HMs and PPs. However, several limitations of the study suggest that more research is needed to draw more certain conclusions. Other studies concluded that the use of HMs during pregnancy may be associated with congenital malformations or even with fetal distress (Mabina et al., 1997). Takei et al. reported a case of occipital meningoencephalocele and cerebellar agenesis in a neonate born at 38 weeks of gestation that was associated with the use of *Tripterygium wilfordii* Hook.f. for the treatment of rheumatoid arthritis early in pregnancy (Takei et al., 1997).

There are several clinical trials conducted to determine the clinical effects of HMs in pregnant women, but only a few of them can be classified as high-quality, randomized controlled trials (RCTs). **Table 2** summarizes the main characteristics of some of the clinical trials investigating the clinical effects of HMs in pregnant women (Dante et al., 2013). Of the 17 clinical trials described here, only 8 studied the efficacy of HMs in pregnant women. Eleven clinical trials explored the effects of ginger (*Z. officinale* Roscoe) (Fischer-Rasmussen et al., 1991; Vutyavanich et al., 2001; Keating and Chez, 2002; Willetts et al., 2003; Sripramote and Lekhyananda, 2003; Smith et al., 2004; Chittumma et al., 2007; Pongrojpaw et al., 2007; Ensiyeh and Sakineh, 2009; Ozgoli et al., 2009; Sharifzadeh et al., 2018), one studied cranberry (*Vaccinium macrocarpon* L.) (Wing et al., 2008), one investigated raspberry leaf in tablets (*R. idaeus* L.) (Simpson et al., 2001), one reviewed garlic (*A. sativum* L.) (Ziaei et al., 2001), one evaluated the use of St. John’s Wort (*H. perforatum* L.) (Samadi et al., 2010), one clinical trial explored the effects of chamomile (*M. chamomilla* L.) (Zafar et al., 2016), and another clinical trial investigated peppermint aromatherapy (*M. piperita* L.) (Joulaeerad et al., 2018).

**Table 2:** Clinical trials on safety and/or efficacy of herbal products in pregnancy.

Herbal medicine	Study design	Number of subjects	Dose/duration	Main findings
Ginger	Double-blind randomized cross-over	N = 15 treatment N = 15 lactose	1 g/day 4 days	Better than placebo on nausea and vomiting. All infants were without deformities and discharges in good conditions (Fischer-Rasmussen et al., 1991).
	Double-blind randomized controlled	N = 35 treatment N = 32 placebo	1 g/day 4 days	Better than placebo on nausea and vomiting. No increase of number of spontaneous abortions, term deliveries, and number of cesareans. No recognized congenital anomalies (Vutyavanich et al., 2001).
	Double-blind randomized controlled	N = 14 treatment N = 11 placebo	1 g/day 2 weeks	Better than placebo on nausea and vomiting. Viable infants at term without major complications (Willetts et al., 2003).
	Double-blind randomized controlled	N = 48 treatment N = 51 placebo	6 g/day 4 days	Better than placebo on nausea and retching. Equal to placebo on vomiting. No increased risk of fetal abnormalities, low birthweight and Apgar scores compared with general population of infants born at the same hospital and year (Willetts et al., 2003).
	Single-blind randomized controlled	n = 32 treatment n = 35 placebo	1 g/day 4 days	Better than placebo on nausea and vomiting (Ozgoli et al., 2009).
	Double-blind randomized controlled	N = 64 treatment N = 64 vitamin B_6_	1.5 g/day Vitamin B_6_: 30 mg/day 3 days	Equal to vitamin B_6_ on nausea and vomiting. (Sripramote and Lekhyananda, 2003)
	Double-blind randomized controlled	N = 146 treatment N = 145 vitamin B_6_	1.05 g/day Vit B_6_: 75 mg/day 3 weeks	Equal to vitamin B_6_ on nausea, retching, and vomiting. No differences in number of congenital abnormalities or other birth outcomes (gestational age, live births, abortions, stillbirths, birth weight) between groups (Smith et al., 2004).
	Double-blind randomized controlled	N = 61 treatment N = 62 vitamin B_6_	1.95 g/day Vitamin B_6_: 25 mg/day 4 days	Better than vitamin B_6_ on nausea and vomiting (Chittumma et al., 2007).
	Triple-blind randomized controlled	N = 28 treatment N = 26 vitamin B_6_ N = 23 placebo	500 mg BID 4 days 40 mg BID 4 days 4 days	Ginger is more effective than placebo at reducing nausea and vomiting; however, vitamin B_6_ was also effective compared to placebo. (Chittumma et al., 2007; Sharifzadeh et al., 2018)
	Double-blind randomized controlled	N = 35 treatment N = 34 vitamin B_6_	1 g/day Vitamin B_6_: 40 mg/day 4 days	Better than vitamin B_6_ on nausea. Equal to vitamin B_6_ on vomiting. No increase of number of spontaneous abortions, term deliveries, and number of cesareans. No recognized congenital anomalies (Ensiyeh and Sakineh, 2009).
	Double-blind randomized controlled	N = 77 treatment N = 74 dimenhydrimate	1 g/day dimenhydrimate: 100 mg 1 week	Equal to dimenhydrimate on nausea and vomiting (Pongrojpaw et al., 2007).
Cranberry	Randomized, controlled	N = 58 treatment A N = 67 treatment B N = 63 placebo	A: 240 mg/day B: 80 mg/day Until delivery	No statistically significant difference between the treatments. No differences on neonatal outcomes between the groups (Wing et al., 2008).
Raspberry leaf	Double-blind randomized controlled	N = 96 treatment N = 96 placebo	2.4 g/day From 32 weeks to labor	No statistically significant difference in duration of labor, length of gestation period, medical augmentation of labor, need for epidural block, or cesarean section rate (Simpson et al., 2001).
Garlic	Single-blind randomized controlled	N = 50 treatment N = 50 placebo	800 mg/day 8 weeks	No statistically significant difference in the means of HDL, LDL, triglyceride, inhibition of platelet aggregation, means of systolic and diastolic blood pressure. Statistically significant difference in the means of total cholesterol and hypertension alone. (Ziaei et al., 2001)
Chamomile	Double-blind randomized controlled	N = 42 treatment N = 47 Pentazocine N = 42 placebo	1 ml of saline + 3 drops of 1 M solution of chamomile 1 ml of Pentazocine (30 mg) + oral placebo 1 ml of saline + oral placebo	No adverse events were noted. Chamomile and Pentazocine did not significantly differ from placebo (Zafar et al., 2016).
Peppermint		N = 28 treatmentN = 28 placebo	10%	There were no statistical significant difference in nausea symptoms between groups; however, improvement in symptoms were observed in both treatment and placebo arms (Joulaeerad et al., 2018).
St John’s Wort	Double-blind randomized controlled	N = 47 treatment N = 44 placebo N = 34 control	Oily extract 16 days	Statistically significant difference in wound healing and scar formation between the three groups (Samadi et al., 2010).

HDL, high-density lipoporotein; LDL, low-density lipoporotein.

The most studied HM was ginger. From the 11 RCTs described, 6 investigated ginger compared to placebo (Fischer-Rasmussen et al., 1991; Vutyavanich et al., 2001; Keating and Chez, 2002; Willetts et al., 2003; Ozgoli et al., 2009; Sharifzadeh et al., 2018), 4 to pyridoxine (vitamin B_6_) (Sripramote and Lekhyananda, 2003; Smith et al., 2004; Chittumma et al., 2007; Ensiyeh and Sakineh, 2009), and 1 to dimenhydrinate (Pongrojpaw et al., 2007). When compared to placebo, all trials showed superiority of ginger in the management of nausea and vomiting except one (Willetts et al., 2003). When compared to vitamin B_6_, results were not homogeneous. No differences were found between ginger and dimenhydrinate groups in controlling either nausea or vomiting.

Few RCTs were conducted to study other HM efficacy and safety in pregnant women. Only one RCT was identified exploring the effects of cranberry compared with placebo in the prevention of UTIs among pregnant women (Wing et al., 2008). The incidence of UTIs or neonatal outcomes were not different between groups; however, this study did not have enough power to detect such a difference. The Cochrane Collaboration reviewed the use of cranberry for the prevention and treatment of UTIs in women in 2008 (Jepson and Craig, 2008). The review concluded that cranberry use significantly reduced the incidence of symptomatic UTIs, but pregnant women were not included in these studies. Another RCT investigated the use of raspberry leaf during pregnancy (Simpson et al., 2001). Consumption of raspberry leaf tablets from 32 weeks of pregnancy until labor resulted in a shorter second stage of labor and in a decrease of the rate forceps delivery when compared to the control group. Consumption of raspberry leaf tablets had no adverse effects on mothers and newborns. A randomized, single-blind, placebo clinical trials investigated the effects of garlic tablets (800 mg/day) taken by pregnant women during the third trimester of pregnancy on plasma lipids, platelet aggregation, and the ability to prevent preeclampsia (Ziaei et al., 2001). The authors did not find significant differences in plasma high-density lipoprotein, low-density lipoprotein, and triglyceride levels or inhibition of platelet aggregation between case and control groups. Garlic treatment did not prevent preeclampsia, but did reduce total cholesterol levels and incidence of hypertension in pregnant women (Ziaei et al., 2001). It has been suggested that high cholesterol levels as well as gestational or chronic hypertension are associated with negative effects on mother and fetus health (Bartels and O’Donoghue, 2011; Mustafa et al., 2012). Therefore, reduction of cholesterol and blood pressure levels may lead to the reduction of unfavorable pregnancy outcomes. Samadi et al. investigate the effects of St. John’s Wort ointment on cesarean wound healing and on the formation of hypertrophic scars in Iranian women giving birth by cesarean section (Samadi et al., 2010). It was concluded that St. John’s Wort ointment application enhanced wound healing and reduced hypertrophic scar formation. Zafar et al. investigated the efficacy of chamomile as a labor analgesic in a randomized, placebo controlled, double-blind RCT (Zafar et al., 2016). No significant analgesic or adverse effects were observed between group using chamomile and control group. Similarly, Joulaeerad et al. studied the effectiveness of peppermint aromatherapy on pregnancy-related nausea and vomiting in a single-blind RCT (Joulaeerad et al., 2018). Investigators did not observe significant reduction in nausea and vomiting in women receiving peppermint aromatherapy compared with the placebo group.

Overall, data regarding the safety and efficacy of many HMs commonly used during pregnancy are limited. HMs used in clinical trials were not always standardized to bioactive constituents and often have limited quality control. This lack of standardization makes conclusions sometimes controversial, with no specific recommendations about herbal use by pregnant women. The duration of the treatment is usually very short, and most of them are small in scale, often 10–15 women. It is difficult to estimate a specific number of individuals needed to be recruited, as significance depends on some other factors. We believe that further studies should be performed with larger samples sizes (n = 30–50 per groups) and studies should include all trimesters to make these determinations. However, these clinical trials should take in consideration data received in preclinical *in vivo* or *in vitro* studies of HMs. In our opinion, it will be unethical to conduct clinical trials of HMs with potential embryotoxicity in pregnant women. Additionally, treating physicians need to be aware of potential embryotoxicity of certain HMs and advise against use of these HMs by pregnant women.

## Herbal-Drug Interactions

### Epilepsy in Pregnancy and HM Use

It is not uncommon for women with epilepsy to be of a childbearing age. There is little evidence substantiating the use of herbals for the treatment of seizures. In fact, the use of certain HM may increase the frequency of seizures, cause side effects or interact with conventional AEDs. One herbal product (extracts of the *Cannabis sativa* L. plant) has been approved by the Food and Drug Administration for treating certain seizure types (HIGHLIGHTS OF PRESCRIBING INFORMATION).

Cannabidiol extracted from *C. sativa* has been approved for use in certain types of seizure disorders. Although extracts of the cannabis plant have been readily available in some countries, they have not been accessible for patients in the United States until recently. There is a broad spectrum of products with some including other cannabinoids such as tetrahydrocannabiol. Use of cannabis during pregnancy has been shown to be associated with adverse outcomes for both infant and mother (Gunn et al., 2016). *In vitro* studies indicate that cannabidiol inhibits several cytochrome enzymes including CYP3A4, CYP3A5, and CYP2D6 (Yamaori et al., 2011a; Yamaori et al., 2011b), but induces CYP1A1 (Yamaori et al., 2015). Additionally, in an *in vitro* study, cannabidiol has been shown to induce UDP-glucuronosyltransferases UGT1A9 and UGT2B7 (Al Saabi et al., 2013). This could lead to complex interactions with AEDs metabolized by these pathways, increasing or decreasing their plasma levels increasing the risk of therapeutic failure or adverse effects. Cannabidiol has been reported to have interactions with several AEDs including clobazam, rufinamide, topiramate, zonisamide, and eslicarbazepine (Gaston et al., 2017).

As there is little evidence available for the use of other HMs in epilepsy patients, there is even less accessible to support use in pregnancy. Some AEDs exhibit complicated pharmacokinetics, and additional medications or HMs have the potential to interact leading to the potential to effect therapy. Some commonly used HMs by patients with epilepsy are St. John’s Wort (*H. perforatum* L.), Gingko (*Gingko* biloba L.), Borgae (*Borago officinalis* L.), Valerian (*V. officinalis* L.), and Cannabis (*C. sativa* L.) (Spinella, 2001; Liu et al., 2017).

St. John’s Wort (*H. perforatum* L.) is used as a complementary medicine for mild depression (Linde et al., 1996; Kim et al., 1999; Linde and Mulrow, 2000) and some psychiatric indications (Martinez et al., 1994; Kasper, 1997; Taylor and Kobak, 2000). There is some evidence for minimal risk of St. John Wort’s use in pregnancy (Grush et al., 1998). Preclinical studies in animals demonstrated that exposure to this HM during pregnancy does not impair cognitive function or have any long-term adverse effects on fetus (Rayburn et al., 2000; Rayburn et al., 2001). However, there are several interactions of St. John’s Wort with AEDs (Wang et al., 2001; Schulz, 2001; Henderson et al., 2002; Markowitz et al., 2003; Durr et al., 2000). These interactions involve induction/inhibition of cytochrome P450 enzyme systems that metabolize AEDs, leading to toxicity from overexposure or reduced efficacy from sub-therapeutic levels. Based on an *in vitro* data it is predicted that St John’s Wort reduces concentrations of CYP3A4 substrates (Komoroski et al., 2004). However, it does not seem to further induce carbamazepine clearance after 14 days of treatment in healthy volunteers (Burstein et al., 2000).

Gingko, an extract from the leaves and seeds of the plant *G. biloba* L. has been used in the treatment of Alzheimer’s (Kanowski et al., 1996; Oken et al., 1998) and premenstrual syndrome (Tamborini and Taurelle, 1993). It is reported to be pro-convulsive in animal models (Pilija et al., 2004) and initiates seizures in children (Fessenden et al., 2001). Gingko has been considered relatively safe (Hashiguchi et al., 2015), however caution should be used during lactation, as it partitions into breast-milk (Romaszko et al., 2014).

Flowers of *B. officinalis* L. containing alkaloids, especially pyrrolizidine, are consumed as an herbal tea. The AED, phenobarbital causes structural changes in pyrrolizidine chemistry, rendering it carcinogenic and teratogenic (Kast, 2001; Roeder et al., 2015). Pyrrolizidine has also been implicated in inducing premature labor by behaving as a prostaglandin E agonist (Kast, 2001).

Root extracts prepared from *V. officinalis* L. are used for the treatment of insomnia (Ziegler et al., 2002) and anxiety (Kohnen and Oswald, 1988). Although valerian has been implicated in the inhibition of CYP3A4 and P-glycoprotein (Hellum and Nilsen, 2008), no clinically relevant interactions with medications metabolized by these enzymes have been observed (Donovan et al., 2004).

In summary, management of epilepsy in pregnancy can be complicated and challenging due to changing physiology and pharmacokinetics. HMs have the potential to interact with the AED therapy leading to reduced efficacy and increased complications during pregnancy increases the risk to both mother and fetus. Healthcare providers should counsel women with epilepsy about the risks of concurrent use, especially during pregnancy.

### Hypertension in Pregnancy and HM Use

Hypertensive disorders of pregnancy include chronic hypertension, gestational hypertension, preeclampsia, and chronic hypertension with superimposed preeclampsia (Lai et al., 2017). Hypertension can be one serious complication of pregnancy leading to potential mortality and morbidity of the mother and fetus (Mustafa et al., 2012). Nowadays, the frequency of preeclampsia defined as a new onset of hypertension accompanied by the proteinuria occurring after 20 weeks of gestation (Mustafa et al., 2012; Townsend et al., 2016) is on the rise due to the increasing number of older women or women with comorbidities becoming pregnant (Townsend et al., 2016). Preeclampsia can significantly contribute to premature labor and maternal mortality (Townsend et al., 2016). Additionally, preeclampsia may affect multiple organs and systems resulting in impairment of the cardiovascular (e.g., acute respiratory distress syndrome, cardiomyopathy), neurons (e.g., eclampsia, cerebral thrombosis, and hemorrhage), renal and hepatic systems (acute kidney injury, glomerular endotheliosis, hepatic periportal inflammation, acute fatty liver of pregnancy).

Antihypertensive therapy during pregnancy is aimed to reduce the risk of severe hypertension and subsequent morbidities (e.g., renal injury). Labetalol is a first-line antihypertensive therapy recommended for the treatment of the moderate hypertension (Committee on Obstetric P, 2017). Methyldopa and nifedipine can be used as alternative drugs, while the use of angiotensin-converting enzyme drugs is not recommended in pregnancy (Committee on Obstetric P, 2017). Recent evidence-based recommendations on the treatment of acute onset of severe hypertension in pregnancy were released by the American College of Obstetricians and Gynecologists in 2017 (Committee on Obstetric P, 2017). Immediate release oral nifedipine was recommended as first-line therapy for pregnant women with acute onset of hypertension. Intravenous administrations of labetalol or hydralazine were also suggested (Committee on Obstetric P, 2017).

Labetalol and nifedipine are the two most commonly used drugs during pregnancy. Nifedipine is metabolized by the CYP3A4 enzyme and eliminates from the human body as pyridine and carboxylic acid of nifedipine (Zisaki et al., 2015). In the human body, labetalol undergoes extensive metabolic changes in the gut and liver (MacCarthy and Bloomfield, 1983). Glucuronidation, mainly by UGT1A1 and UGT2B7, is the primary pathway of labetalol elimination (Jeong et al., 2008). The drug is metabolized by the UDP glucuronosyltransferases into two pharmacologically inactive metabolites, alcoholic glucuronide, and o-phenyl glucuronide (MacCarthy and Bloomfield, 1983). The metabolism of these drugs can be potentially influenced by HMs concurrently taken by pregnant women with or without clinician’s permission.

Pregnant women may use HMs as complementary or alternative medicine to reduce their blood pressure. Many women taking HMs see them as a less-toxic natural alternative to prescription drugs. Therefore, they may consider taking Rauwolfia [*Rauvolfia serpentina (L.)* Benth. ex Kurz], buchu [*Agathosma betulina* (P.J.Bergius) Pillans], garlic (*A. sativum* L.), black or green tea [*C. sinensis* (L.) Kuntze], or Roselle (*Hibiscus sabdariffa* L.), hypertensive HMs (Tabassum and Ahmad, 2011; Lobay, 2015). For many of HMs, antihypertensive properties were established in animal models (Tabassum and Ahmad, 2011). Rauwolfia is a well-known HM used for the treatment of hypertension (Lobay, 2015). Reserpine isolated from this herb was approved and use as a conventional drug for hypertension treatment. Currently, rauwolfia extracts may be used as adjunct therapy to the prescription medication (Lobay, 2015). Below we discuss potential interactions of HMs with prescription drugs used for the treatment of hypertension in pregnant women (**Table 3**).

**Table 3:** Potential HM interactions with conventional antihypertensive drugs taken by pregnant women.

Conventional drugs	Conventional drug metabolism	Herbal medicine	HM potential effect on the conventional drug metabolism
Nifedipine	CYP3A4	*Rauvolfia serpentina* (L.) Benth. ex Kurz	↓ CYP3A4 (Flynn and Vohra, 2018), clinical implications not known
		*Valeriana officinalis L.*, *Camellia sinensis* (L.) Kuntze, *Hibiscus sabdariffa* L.	↓ CYP3A4 (Lefebvre et al., 2004; Johnson et al., 2013; Tam et al., 2014; Sprouse and van Breemen, 2016) Insignificant herb-drug interaction in humans
		*Hypericum perforatum* L.,	↑ CYP3A4 (Henderson et al., 2002; Hellum and Nilsen, 2008; Sprouse and van Breemen, 2016) Confirmed in preclinical and clinical studies
		Ginkgo biloba L.	No significant changes PK parameters in volunteers. Atypical 2-fold increase plasma concentrations in 2 patients (Yoshioka et al., 2004).
		*Silybum marianum* (L.) Gaertn.	Not potent inhibitor of CYP3A4 in humans (Fuhr et al., 2007).
		Grapefruit juice	May inhibit metabolism and gastric emptying (Odou et al., 2005).
Labetalol	UGT1A1 UGT2B7	*Panax* spp. *Echinacea* spp. *Silybum marianum* (L.) Gaertn. *Valeriana officinalis L. Camellia sinensis* (L.) Kuntze	↓ UGT1A1 (Mohamed et al., 2010), clinical implications not known

An *in vitro* study conducted by Flynn et al. demonstrated that rauwolfia extract and plant alkaloids may inhibit CYP3A4 activity (except yohimbine) and significantly increase glucuronosyltransferase activity (except corynanthine and reserpic acid) (Flynn and Vohra, 2018). However, clinical implications of this study are not known. It has been also shown that valerian can inhibit CYP3A4 and P-glycoprotein activities in preclinical studies (Lefebvre et al., 2004; Sprouse and van Breemen, 2016). However, CYP3A4 inhibition demonstrated in preclinical studies was not confirmed clinical studies (Sprouse and van Breemen, 2016). St. John’s Wort is considered to be a potent enhancer of CYP3A4 in vitro (Hellum and Nilsen, 2008). This preclinical observation was confirmed by clinical trials (Sprouse and van Breemen, 2016). Moore L. et al. have found that St. John’s Wort’s extracts increased expression of CYP3A4 in human hepatocytes *via* activation of pregnane X receptors (Moore et al., 2000). Green and black leisure teas were capable of inhibiting CYP3A4 enzyme activity *in vitro* (Tam et al., 2014; Sprouse and van Breemen, 2016). Ethanolic extract of hibiscus inhibited the activity of nine CYP450 enzymes including CYP3A4 (Johnson et al., 2013). Nevertheless, interactions of leisure teas and ethanolic hibiscus extract with drugs metabolized by CYP3A4 were considered to be clinically irrelevant herb-drug interactions (Donovan et al., 2004; Gurley et al., 2005; Hellum and Nilsen, 2008; Johnson et al., 2013).

Concurrent administration of an extract of ginkgo leaves with nifedipine in rats increased the area under the concentration-time curve and the absolute bioavailability of nifedipine, demonstrating the potential interactions with nifedipine in rats (Yoshioka et al., 2004). Concurrent administration of ginkgo with nifedipine to volunteers did not change significantly pharmacokinetic parameters of nifedipine (Yoshioka et al., 2004). However, two subjects had significantly increased levels of plasma nifedipine, severe long lasting headaches, and hot flushes. Authors have concluded that ginkgo and nifedipine should not be used concurrently (Yoshioka et al., 2004). An *in vitro* studies have demonstrated that bioactive constituents present in milk thistle [*Silybum marianum* (L.) Gaertn.] extract inhibited CYP3A4 enzyme (Fuhr et al., 2007). Simultaneous administration of milk thistle extract with nifedipine to human volunteers did not significantly change the metabolism of nifedipine. Some tendency to delayed absorption of the drug was observed. It has been concluded that milk thistle is not potent inhibitor of CYP3A4 inhibitor in humans (Fuhr et al., 2007). Grapefruit (*Citrus × paradisi* Macfad.) juice taken with nifedipine can inhibit drug metabolism and delay gastric emptying (Odou et al., 2005).

According to Mohamed et al. several HMs can inhibit UGT1A1 activity, specifically ginseng (*Panax* spp.), coneflower (*Echinacea* spp.), milk thistle (*S. marianum* L.), valerian (*V. officinalis* L.), and green tea epigallocatechin gallate (Mohamed et al., 2010). These herbs, when concurrently taken with labetalol, can potentially interfere with its metabolism.

In summary, hypertension occurring during pregnancy is a serious condition that may result in life-threatening complications. There is not enough evidence to conclude that medicinal herbs that women can consume during pregnancy will have a clinically meaningful effect on the pharmacokinetics and pharmacodynamics of anti-hypertensive medications. Based on the currently available data, we believe that concurrent use of labetalol and nifedipine with HMs in recommended doses would not alter metabolism of conventional drugs. However, many consumers consider HMs as a safe alternative to the conventional drugs and may exceed recommended HM doses. In this situation, the concurrent use of HM may complicate treatment of hypertension in pregnancy. The most alarming is the clinically proven interaction of nifedipine with St. John’s Wort. Therefore, healthcare professionals should discuss with their patients potential risks of the concomitant use of anti-hypertensive drugs and herbal supplements.

### Asthma in Pregnancy and HM Use

Asthma is a disorder defined by the inflammatory narrowing of the bronchial tubes of the lungs and is the most common chronic condition associated with pregnancy (Murphy and Schatz, 2014), affecting an estimated 4%–8% of pregnant women (National Heart Lung et al., 2005). Poor asthma control during pregnancy is associated with adverse maternal outcomes (e.g., hypertension, gestational diabetes, preterm delivery, maternal mortality) and neonatal outcomes (e.g., congenital malformation, hospitalization, and death) (Murphy and Schatz, 2014; Baghlaf et al., 2017; McLaughlin et al., 2018). The U.S. Department of Health and Human Services recommendations prioritize asthma control over possible pharmaceutic side effects related to pregnancy (National Heart Lung et al., 2005), as it has been shown that effective perinatal control of asthma has been associated with improved outcomes for both mother and infant (Ibrahim et al., 2018; Murphy and Schatz, 2014; Al Ghobain et al., 2018; McLaughlin et al., 2018).

Asthma therapy during pregnancy is mainly aimed at maintaining airways, reducing the use of short-acting inhaled beta_2_-agonists, and reducing the risk of acute exacerbations to preserve oxygenation for both mother and offspring. Current asthma daily therapy commonly includes a short-acting beta_2_-agonist (e.g., albuterol) and depending on severity, an inhaled corticosteroid (e.g., fluticasone, budesonide, beclomethasone, or mometasone), a long-acting beta-agonist (e.g., salmeterol, formoterol), or a leukotriene receptor antagonist (e.g., montelukast, zafirlukast). Additional medications may include systemic corticosteroids (e.g., prednisone, prednisolone, or methylprednisolone), a 5-lipoxygenase inhibitor (e.g., zileuton), an immunomodulator (Omalizumab), cromolyn, or a methylxanthine (e.g., theophylline) (National Heart Lung et al., 2005; Namazy and Schatz, 2018).

Despite evidence suggesting improved outcomes for both mother and offspring, women will often alter or stop their asthma treatment regimen. Recently, (Ibrahim et al., 2018; Ghobain et al., 2018) have reported that over half of women have uncontrolled asthma during their pregnancy and that 40%–50% of women believe that asthma medications, corticosteroids, in particular, will be more harmful to their offspring than uncontrolled asthma. Conceivably, these women could be supplementing with HMs and dietary supplements to help control their asthma symptoms, ameliorate discomfort associated with pregnancy, or improve general health.

Depending on the metabolic paths of these medications, HMs may result in unintended patient consequences (**Table 4**); however, at present, the clinical significance of herb-drug interactions associated with asthma medications is generally unknown. Of particular importance are inhaled corticosteroids (e.g. budesonide [AstraZeneca, 2006], mometasone [Merck Sharp and Dohme Corp, 2018] fluticasone [GlaxoSmithKline, 2017] beclomethasone [Teva Respiratory, 2017], and corticosteroids (e.g., prednisolone, methylprednisolone) [Bergmann et al., 2012; Pfizer, 2018]+, which are foundational treatment for asthma, are undergo significant metabolism by CYP3A4. Montelukast is another example where herbal supplementation may lead to unintended affects. Montelukast is metabolized by CYP2C8 as well as CYP3A4 at clinically relevant concentrations [Merck Sharp and Dohme Corp, 2016].

**Table 4:** Potential HM interactions with conventional asthma control drugs taken by pregnant women.

Conventional drug	Conventional drug metabolism	Herbal medicine	Potential effect
Corticosteroids (e.g. Fluticasone, Budesonide, Beclomethasone, Prednisone, Prednisolone)	CYP3A4	*Trachyspermum ammi* (L.) Sprague	CYP3A4 inhibitor and blood thinner (Bairwa et al., 2012)
		*Angelica dahurica* (Hoffm.) Benth. & Hook.f. ex Franch. & Sav	CYP3A4 inhibitor (Ishihara et al., 2000)
		*Eucalyptus globulus* Labill.	CYP3A4 inhibitor (Unger and Frank, 2004)
		St. John’s Wort	CYP 3A4 inducer (Henderson et al., 2002)
Montelukast	OATP2B1 substrate	Orange juice	↓ AUC (*SLCO2B1 c.935G/G homozygote)* (Mougey et al., 2011; Chen et al., 2018)
	CYP3A4	Grapefruit juice	↑ AUC (Cingi et al., 2013)
		*Eucalyptus globulus* Labill.	CYP3A4 inhibitor (Unger and Frank, 2004)
		*Epigallocatechin gallate*	Extension of systemic half-life (Misaka et al., 2013)
		St. John’s Wort	CYP 3A4 inducer (Komoroski et al., 2004)

Several HMs have been evaluated for roles in asthma control. For example, flavonoids, such as *Sophora flavescens* Aiton (Yang et al., 2013) and *Glycyrrhiza uralensis* Fisch. ex DC. (Yang et al., 2013), have been implicated in the possible prevention of asthma symptoms (Tanaka and Takahashi, 2013) and to possibly reduce the risk of asthma in infants born to mothers with asthma (Lopez-Exposito et al., 2015). Similarly, *Angelica dahurica* (Hoffm.) Benth. & Hook.f. ex Franch. & Sav. root extract (Lee et al., 2011) and green tea (*C. sinensis* (L.) Kuntze) (Yang et al., 2018), have been implicated with anti-asthmatic effects and reduction in asthma-related inflammation, respectively. Eucalyptus oil has also been used for respiratory indications (Ahmed et al., 2017) and it is reported that cineole is associated with improved lung function (Worth and Dethlefsen, 2012). These and other such observations may encourage pregnant women to replace or supplement diet and asthma treatment regimens, despite known interactions with conventional drugs (Smeriglio et al., 2014).

Epigallocatechin gallate, a constituent of teas, has shown *in vitro* inhibition of Cytochrome P450 enzymes, including CYP1A2, CYP2C8, CYP2C9, and CYP3A4 (Albassam and Markowitz, 2017) and therefore has potential effects on the majority of conventional asthma drugs. Similarly, eucalyptus oil (*Eucalyptus globulus* Labill.) was shown *in vitro* to inhibit CYP3A4 (Unger and Frank, 2004). Recently, epigallocatechin gallate was implicated in increasing the systemic half-life of Montelukast (Misaka et al., 2013). *A. dahurica* (Hoffm.) Benth. & Hook.f. ex Franch. & Sav. has been associated with inhibition of CYP3A, as well as CYP2C and CYP2D1 (Ishihara et al., 2000), which may reduce systemic elimination of corticosteroids.

As described in the previous sections, St. John’s Wort is a significant CYP3A4 inducer and therefore may reduce concentrations of both corticosteroids and Montelukast. Pregnant women may also take the antioxidants vitamins C, found in a variety of citrus fruits including grapefruit. Grapefruit is a known inhibitor of CYP3A4 (Mouly et al., 2017) and therefore may interact with glucocorticoids increase systemic exposure. Carum Ajowan [*Trachyspermum ammi* (L.) Sprague] may also be taken orally by pregnant women to treat indigestion or asthma (Bairwa et al., 2012). Ajowan (*Carum copticum* L) is a CYP3A4 inhibitor (Di Minno et al., 2017). Similar to grapefruit it may increase systemic exposure to glucocorticoids. Unripe ajwain contains chromone, a constituent with anticoagulant properties (Boskabady et al., 2014). Use of this herb by pregnant women may result in an excessive bleeding during cesarean section or labor. Montelukast also undergoes carrier mediated transport by OATP2B1. There is some evidence to suggest that grapefruit juice (Cingi et al., 2013), a known CYP3A4 inhibitor, increases montelukast exposure while orange juice (Mougey et al., 2011; Chen et al., 2018) decreases montelukast exposure in individuals with specific OATP2B1 variations (e.g. *SLCO2B1 c.935G/G*).

Poorly controlled asthma during pregnancy is of serious health concern for both mother and offspring, resulting in increased morbidity and mortality. Pregnant women may be supplementing or replacing prescribed conventional drugs with medicinal herbs and dietary supplements. Definitive research into the clinical implications for potential asthma herb-drug interactions has not been completed; however, metabolism of conventional drugs and HMs are often regulated by Cytochrome P450 enzymes [e.g., CYP3A4 (AstraZeneca, 2006; Bergmann et al., 2012; Rajapakse et al., 2016; GlaxoSmithKline, 2017; Teva Respiratory, 2017; Merck Sharp and Dohme Corp, 2018; Pfizer, 2018, CYP2C8 (Yang et al., 2018), CYP2C9 (DEY, 2007; Cornerstone Therapeutics Inc, 2012; AstraZeneca Pharmaceuticals, 2013, CYP2C19 (DEY, 2007), CYP1A2 (Cornerstone Therapeutics Inc, 2012), etc.] or have potential additive effects (**Table 3**). The clinical significance of these observations requires additional study; however, clinicians should be aware of the concurrent use of asthma-related drugs and herbal/dietary supplements, which may complicate treatment. The authors do not recommend replacement or unmonitored supplementations. The authors do recommend monitoring, further retrospective analyses, and continued education of and discussion with patients, clinicians, and scientists.

## Summary

As outlined in this review, the problem has become the introduction of HMs or other natural products similtaneously with conventional drugs during pregnancy and our lack of knowledge of potential herbal-drug interactions, which can impact fetus or mother. Pregnant women often consume HMs without consulting their healthcare providers. There are many known benefits associated with HMs that can help during or after pregnancy such as increasing milk production, decreasing nausea, easing labor pains, relief of morning sickness, or decreasing flatulence. Many women take HMs when they are pregnant without any concern based on the assumption that since herbals are natural, they do not have any worry that these products will cause harm to them or their unborn babies.

There is a desperate need for education to be provided to pregnant women and their healthcare professionals. We need to move away from the idea of HMs not being harmful. Even conventional drugs that can be bought over the counter have the potential to interact with HMs. For example, if pregnant women are taking aspirin as well as HMs that also have antiplatelet activity (El Haouari and Rosado, 2016), it can lead to an increased risk of bleeding and, in some pregnant women, this can become life threatening. This is an area, where there is limited research, and the use of HMs or potential interactions of these with conventional drugs can lead to unknown impacts on the fetus or cause severe complications to the mother. There is an urgent need for more attention to be paid to the safety of combining conventional drugs and HMs during pregnancy.

HMs and their preparations can potentially interact with conventional drugs reducing or increasing the efficacy of the drugs, and in some disease conditions (e.g., hypertension, asthma, epilepsy) this can lead to life-threatening situations. Due to the overall reluctance to undertake studies during pregnancy, we lack information and in many cases with do not know what the optimal therapeutic dose of a drug that should be given to a pregnant woman in her third trimester is. Without this foundational understanding, determining the optimal treatment may be further complicated if she is also taking an HM that reduces the efficacy of her conventional drugs.

Also, of concern is that the embryotoxic effects of HMs have been poorly studied. Clinical pharmacologists, scientists, and physicians need to be more informed regarding preclinical and clinical study findings of adverse effects related to HMs on the developing fetus.

In recent times, it has been recognized through the establishment of the 21^st^ Century Cures Act that there is a need to undertake research to answer questions that are specific in pregnant and lactating women (An Act: To accelerate the discovery, development, and delivery of 21st century cures, and for other purposes). We are not yet at the point of initiating clinical trials to investigate herbal-drug interactions, but the establishment of the Task Force on Research Specific to Pregnant Women and Lactating Women (NIH, 2018) is a move in the right direction. Continued focus, discussion, and research by invested parties will be necessary as we move forward to addressing potential HM and HM-drug interaction safety concerns.

## Author Contributions

SI prepared materials and wrote the *Introduction* and *Efficacy and Safety of HM Used by Pregnant Women* sections. OA was responsible for materials of the *Consumption of Herbal Medicines by Pregnant Women* section. EE and LK prepared materials and wrote the *Hypertension in Pregnancy and HM Use* section. AB and AK were responsible for the *Epilepsy in Pregnancy and HM Use* section. KJ and VB wrote the *Asthma in Pregnancy and Herbal Medicine Use* section. CS summarized presented in the manuscript materials and wrote abstract and summary statement.

## Conflict of Interest

CS is Specialty Chief Editor in *Frontiers Obstetric and Pediatric Pharmacology Journal*. All other authors have no relevant affiliations or financial involvement with any organization or entity with a financial interest in or financial conflict with the subject matter or materials discussed in the manuscript. This includes employment, consultancies, honoraria, stock ownership or options, expert testimony, grants or patents received or pending, or royalties.
